# Baseline Ang-2 Serum Levels as a Predictive Factor for Survival in NSCLC and SCLC

**DOI:** 10.3390/life12122092

**Published:** 2022-12-13

**Authors:** Asimina Nikolakopoulou, Dimitris Tsakogiannis, Flora Zagouri, Eleni Zografos, Lamprini Tzioga, Grigorios Stratakos, Nikolaos Koulouris, Konstantinos Syrigos, Garyfalia Bletsa

**Affiliations:** 1 1st Department of Respiratory Medicine, Medical School, National and Kapodistrian University of Athens, 11527 Athens, Greece; 2 Research Center, Hellenic Anticancer Institute, 10680 Athens, Greece; 3 Department of Clinical Therapeutics, Alexandra Hospital, National and Kapodistrian University of Athens School of Medicine, 11528 Athens, Greece; 4 Third Department of Internal Medicine, Sotiria Hospital, National and Kapodistrian University of Athens, 11527 Athens, Greece

**Keywords:** angiopoietins, Ang-2, lung cancer, NSCLC, SCLC, prognostic factor

## Abstract

Angiopoietin-2 (Ang-2) has been implicated in the development of several types of cancer, including lung malignancy. In the present study, we examined the impact of Ang-2 serum concentration on the development, dissemination, and 5-year overall survival of NSCLC and SCLC. A total of 99 patients with lung cancer were tested. The OS of NSCLC and SCLC patients was estimated using Kaplan–Meier curves and compared through log-rank test. The median serum level of Ang-2 at baseline in both NSCLC and SCLC patients was significantly higher than that of controls (*p* < 0.0001). The Ang-2 serum concentration was not related to metastasis, neither in NSCLC nor in SCLC cases. The OS was found to be significantly shorter for stage IIIβ NSCLC patients with a high baseline Ang-2 serum concentration (*p* = 0.012), while Cox regression analysis showed that Ang-2 is a significant independent factor for poor prognosis for stage IIIβ NSCLC (hazard ratio = 2.97, 95% CI: 1.05–8.40, *p* = 0.04). The concentration of Ang-2 has no impact on the prognosis of SCLC. Ang-2 could be considered as a significant molecular marker that enables the prediction of NSCLC and SCLC development, and is involved in the poor prognosis of stage IIIβ NSCLC.

## 1. Introduction

Lung cancer is a rapidly evolving type of malignancy, accounting for more than 1.5 million cancer associated deaths worldwide [1,2]. With regards to the male population, it is the most frequent type of cancer followed by prostate and colorectal cancer, while among women rates after breast and colorectal cancer [1,3]. Lung cancer is a heterogeneous disease in terms of clinical presentation and histology. According to the cytological features of tumor cells, lung cancer is classified into two main histological groups, small-cell lung cancer (SCLC) (15%) and non-small cell lung cancer (NSCLC) (85%), that confer different growth, metastasis, and treatment motifs [4,5].

Angiogenesis is the physiological procedure by which new blood vessels and capillaries are generated from pre-existing vasculature during wound healing and embryonic development, while it is considered as the key event for the development of several types of cancer, including lung cancer [6,7,8,9]. Angiogenesis is regulated by a complicated balance of proangiogenic and antiagiogenic factors. The most well-known angiogenic factors are vascular endothelial growth factor (VEGF), platelet-derived growth factor (PDGF), and Angiopoietins [10,11]. In humans, Angiopoietins comprise a small group of three secreted glycoproteins named as Angiopoietin-1 (Ang-1), Angiopoietin-2 (Ang-2), and Angiopoietin-4 (Ang-4) (the mouse ortholog is named as Ang-3), which are ligands of the Tie-2 tyrosine kinase receptor [11]. The role of angiopoietins in lung cancer development has gained increased popularity, whereas the vast majority of studies have focused on the relationship of Ang-2 with the development of NSCLC [11]. Nevertheless, the impact of Ang-2 on SCLC development remains rather vague. Nowadays, it is known that serum levels of Ang-2 are involved in primary lung cancer growth, tumor angiogenenis, lyphangiogenesis, advanced stages of NSCLC, and metastasis [11,12,13,14,15,16,17,18,19,20,21]. Moreover, it is significant to underline that surgery is found to be implicated in alterations in the Ang-2 plasma levels of NSCLC patients as well. In particular, it has been observed that postoperative patients exhibit higher Ang-2 plasma levels in association with their preoperative levels [22,23]. In addition, more invasive surgical methods appear to influence Ang-2 levels in patients with early-stage NSCLC [24].

In recent years, a significant interest has emerged in the prognostic efficacy of Ang-2 in lung cancer. In particular, Park et al. (2007) [16] first reported that NSCLC patients with low serum levels of Ang-2 exhibited a better overall survival than those with higher Ang-2 levels. However, numerous research analyses were followed in order to examine the prognostic value of Ang-2 in NSCLC cases, suggesting that Ang-2 is a considerable marker of poor prognosis of NSCLC [14,25,26,27,28]. On the other hand, little is known concerning the role of Ang-2 in the prognosis of SCLC [17,18].

To this end, the present study focused on the determination of the circulating levels of Ang-2 in lung cancer patients in order to evaluate the prognostic role of Ang-2 in NSCLC and SCLC. Moreover, we examined whether Ang-2 serum concentration differs between distinct lung cancer subtypes as well as between different clinicopathological characteristics including age, gender, smoke, and metastasis.

## 2. Materials and Methods

### 2.1. Study Design and Patients

The study enrolled 99 patients (74 males and 25 females) diagnosed with lung cancer between January 2014 and January 2019 at the Thoracic Diseases General Hospital of Athens ‘Sotiria’. In addition, a total of 55 healthy individuals were also recruited in the retrospective analysis. The mean age of the patients and control group was 64.7 ± 8.9 and 52.8 ± 13.4 years, respectively. Information on patients including age, smoke, gender, type of lung cancer, and metastasis was retrieved from the database of our hospital. In particular, 26 lung cancer patients were diagnosed with small cell lung cancer (SCLC), while 73 patients were diagnosed with non-small cell lung cancer (NSCLC). The study population in both NSCLC and SCLC cases consisted of inoperable patients with locally advanced (Stage IIIβ) and patients with distant metastasis (Stage IV). Patients included in the study were not treated with radiotherapy and did not receive any anticancer therapy prior to the baseline measurement of Ang-2. The patients enrolled in the study were treated based on the existing guidelines at the time of the study. In particular, a total of 57 (57/73) NSCLC and 26 (26/73) SCLC patients received platinum-based chemotherapy as first-line treatment in three week cycles up to six cycles, while 16 (16/73) NSCLC patients were treated with platinum-based chemotherapy in combination with anti–vascular endothelial growth factor (anti-VEGF) 15 mg/kg. Follow-up information regarding the cause of death of the examined patients was obtained through a review of medical records. All patients signed an informed consent form and the study was approved from the research committee of the hospital (approval number 15146/14-07-2017).

### 2.2. Sample Preparation and Ang-2 Level Measurement

Blood samples from lung cancer patients and healthy controls were collected between 12:00 a.m. and 14:00 p.m. Sampling was performed directly into serum vacuum tubes with a clot activator. Tubes remained at room temperature for 20–30 min to enable blood clotting and they were centrifuged at 3000× *g* rpm for 15 min at 8 °C. Immediately after centrifugation, the serum samples were stored at −80 °C until use. Serum samples were collected from lung cancer patients before initiation of treatment and after the third cycle of chemotherapy. Serum samples were tested for Ang-2 concentration at the baseline. The measurement of Ang-2 serum levels was carried out in duplicates through enzyme-linked immunosorbent assay (ELISA), using the Human Angiopoietin—2 Quantikine^®^ *ELISA kit* (R&D Systems, Minneapolis, MN, USA), according to the manufacturer’s instruction. The acquired fluorescence data were analyzed using Multiscan™ FC Microplate Photometer (Thermo Fisher Scientific, Inc., Waltham, MA, USA).

### 2.3. Statistical Analysis

The Ang-2 levels were classified as low or high for each patient using the median value as cut-off. The association of Ang-2 serum levels with the development of lung cancer as well as with the clinicopathological parameters, including age, gender, smoke, metastasis, and lung cancer type, were estimated using the Mann–Whitney U test and Kruskal–Wallis test followed by the Dunn multiple comparison post hoc test. Sensitivity, specificity, and area under the curve (AUC) of the serum level of Ang-2 were calculated using ROC (receiving operator characteristic) analysis. Survival curves were plotted using the Kaplan–Meier method and differences in survival rates were examined using the logrank test. The prognostic significance of each variable to overall survival was estimated through the Cox regression model. The Wilcoxon signed rank test was used to compare Ang-2 levels at the third cycle of chemotherapy. Data were analyzed using the SPSS (version 23) statistic software package (SPSS, Inc., Chicago, IL, USA). *p*-values were considered as statistically significant bellow the 0.05 cut-off level.

## 3. Results

### 3.1. Association of Ang-2 Serum Levels with the Development of Lung Cancer

**Figure 1:**
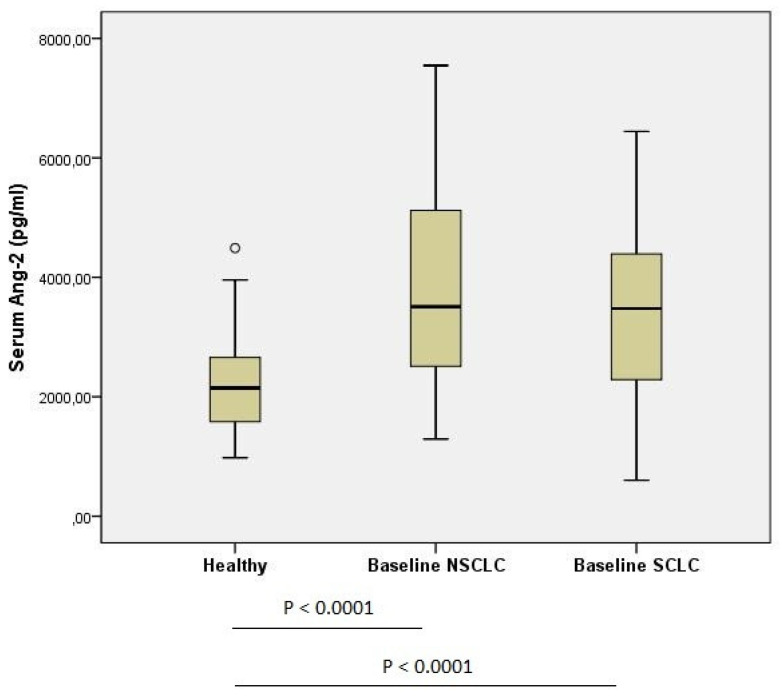
Ang-2 serum levels of healthy controls compared with the Ang-2 serum levels of patients with lung cancer.

Serum levels of Ang-2 were measured in a total of 99 patients along with 55 healthy individuals. The median serum level of Ang-2 at baseline was 3493 pg/mL for lung cancer patients and was significantly higher than that of controls (median concentration; 3493 pg/mL vs. 2148 pg/mL, *p* < 0.0001, Table 1, Figure 1). The most optimal cut-off value of Ang-2 serum concentration was 2547 pg/mL, offering a sensitivity of 72.7% and a specificity of 71% for distinguishing lung cancer patients from healthy controls. The area under the curve (AUC) was 0.779 (95% CI: 0.707–0.852, *p* < 0.0001).

The association of Ang-2 serum levels with the different clinicopathological characteristics of patients was evaluated. No significant differences in Ang-2 levels were reported among the different patient characteristics, including age (*p* = 0.91), smoke (*p* = 0.16), gender (*p* = 0.61), and lung cancer type (*p* = 0.91) (Table 1). Although patients with metastatic disease had higher Ang-2 serum levels when compared with patients with no metastasis, this association failed to reach the limits of statistical significance (median concentration; 3538 pg/mL vs. 3318 pg/mL, *p* = 0.23, Table 1). The correlation of Ang-2 serum concentration with the development of lung cancer was further investigated with respect to the two different histological groups (SCLC, NSCLC). Baseline serum levels of Ang-2 in both SCLC and NSCLC were significantly higher than that of the control group (SCLC: median concentration; 3479 pg/mL vs. 2148 pg/mL, *p* < 0.0001, NSCLC: median concentration; 3508 pg/mL vs. 2148 pg/mL, *p* < 0.0001, Table 2). Moreover, no significant association of Ang-2 levels was reported between Ang-2 levels and presence of metastasis, neither in SCLC nor in NSCLC patients.

### 3.2. Association of Baseline Ang-2 Serum Level with the 5-Year Overall Survival of NSCLC and SCLC

**Figure 2:**
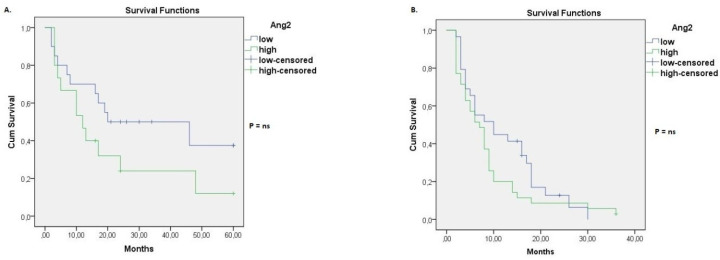
Kaplan–Meier curve for overall survival grouped by baseline serum Ang-2 levels. (**A**) Overall survival for stage ΙΙΙβ lung cancer patients with high or low serum Ang-2 at baseline. (**B**) Overall survival for stage IV lung cancer patients with high or low serum Ang-2 at baseline.

Kaplan–Meier curves and logrank test were conducted in order to examine the impact of baseline Ang-2 serum level on the overall survival of lung cancer patients diagnosed as stage IIIβ and IV, respectively. According to our results, Ang-2 baseline levels were not associated with overall survival in stage IIIβ lung cancer patients (Figure 2A). No significant association was observed in stage IV lung cancer patients as well (Figure 2B). 

**Figure 3:**
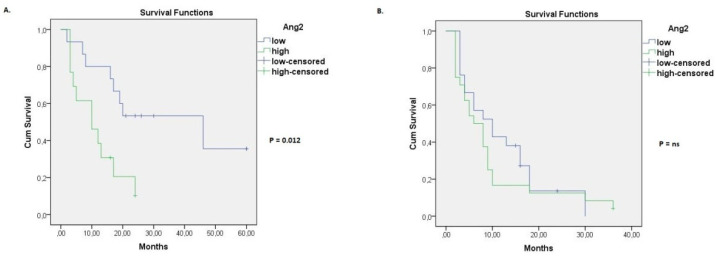
Kaplan–Meier curve for overall survival grouped by baseline serum Ang-2 levels. (**A**) Overall survival for stage ΙΙΙβ NSCLC patients with high or low serum Ang-2 at baseline. (**B**) Overall survival for stage IV NSCLC patients with high or low serum Ang-2 at baseline.

**Figure 4:**
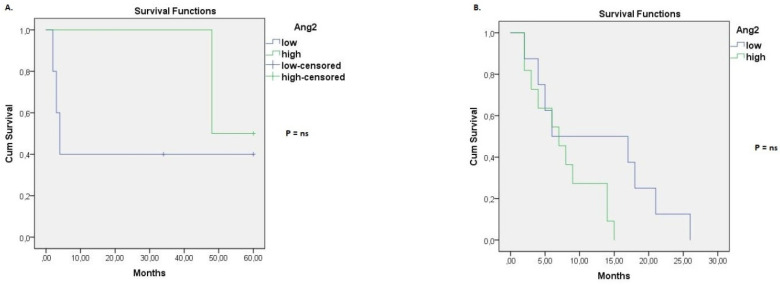
Kaplan–Meier curve for overall survival grouped by baseline serum Ang-2 levels. (**A**) Overall survival for stage ΙΙΙβ SCLC patients with high or low serum Ang-2 at baseline. (**B**) Overall survival for stage IV SCLC patients with high or low serum Ang-2 at baseline.

The effect of baseline Ang-2 level in the survival of patients was also evaluated per histological type of lung cancer (SCLC, NSCLC). Considering stage IIIβ NSCLC, it was found that the median overall survival was significantly shorter for patients with a high baseline Ang-2 serum concentration. In particular, the median overall survival was 10 months in stage IIIβ NSCLC patients with a high Ang-2 baseline level (95% CI: 1.8–18.2), while the median overall survival was 46 months in stage IIIβ NSCLC patients with a low Ang-2 baseline concentration (95% CI: 9.5–82.5) (*p* = 0.012, Figure 3A). Multivariate Cox regression model further revealed that the high baseline Ang-2 concentration is an independent predictor of survival for patients with stage IIIβ NSCLC (hazard ratio = 2.97, 95% CI: 1.05–8.40, *p* = 0.04, Table 3). With regards to stage IV NSCLC patients, median overall survival was 6 months in patients with a high Ang-2 baseline level (95% CI: 2.8–9.2), whereas median overall survival was 10 months in patients with a low Ang-2 baseline level (95% CI: 4.0–15.9). However, these results were not considered as statistically significant (*p* = ns, Figure 3B). Moreover, Ang-2 baseline levels were not associated with overall survival in SCLC patients, neither of stage IIIβ nor of stage IV (Figure 4).

### 3.3. Ang-2 Serum Concentration before Treatment and after the Third Cycle of Chemotherapy

**Figure 5:**
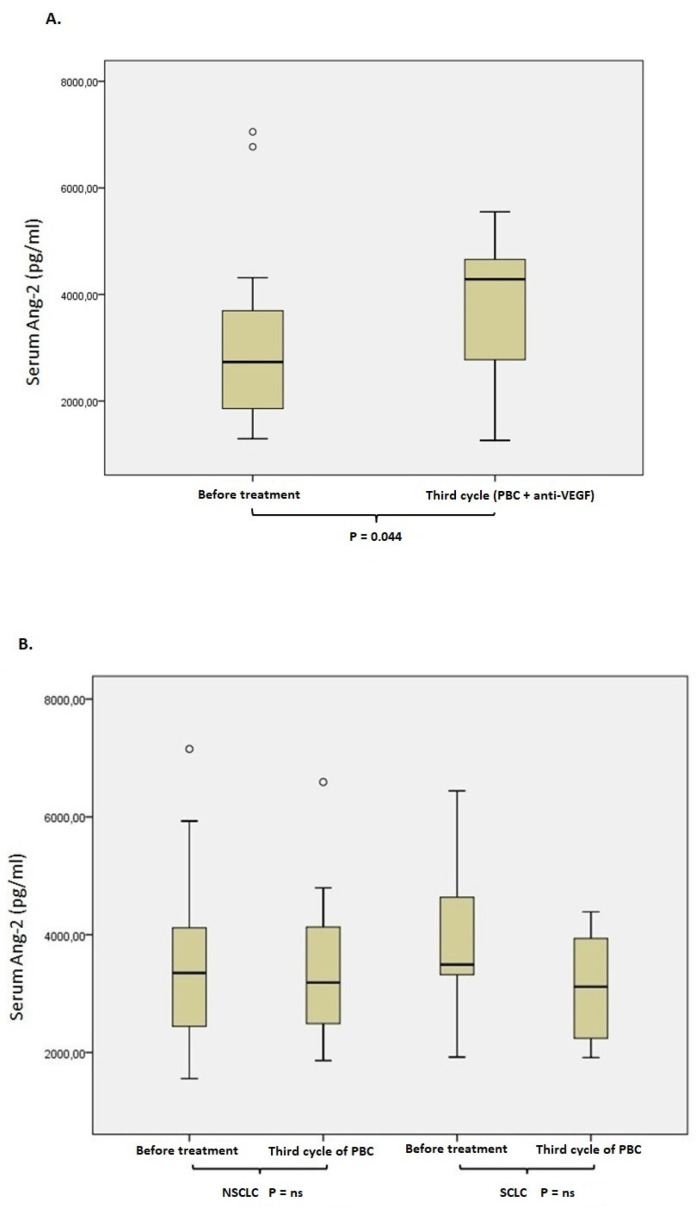
Ang-2 serum levels of patients at the third cycle of chemotherapy. (**A**) Ang-2 serum levels of NSCLC patients who received PBC in combination with anti-VEGF before treatment and after the third cycle of chemotherapy. (**B**) Ang-2 serum levels of SCLC patients who received PBC before treatment and after the third cycle of chemotherapy.

Differences between the Ang-2 concentration before treatment and after the third cycle of chemotherapy were also evaluated in cases where blood samples were available. In particular, the median Ang-2 level in NSCLC patients (n = 16) who received platin-based chemotherapy in combination with anti-VEGF factor was significantly higher after the third cycle of chemotherapy when compared with the Ang-2 serum level at admission (median concentration; 4285 pg/mL vs. 2734 pg/mL, Wilcoxon signed rank test, *p* = 0.044, Figure 5A). In addition, NSCLC patients who received only platin-based chemotherapy (n = 23) provided no significant differences at Ang-2 serum levels after the third cycle of chemotherapy (median concentration; 3188 pg/mL vs. 3350 pg/mL, Wilcoxon signed rank test, *p* = 0.58, Figure 5B). Finally, SCLC patients (n = 13) treated with platin-based chemotherapy showed no significant alterations in Ang-2 serum level after the third cycle of chemotherapy as well (median concentration; 3115 pg/mL vs. 3493 pg/mL, Wilcoxon signed rank test, *p* = 0.54, Figure 5B).

## 4. Discussion

Ang-2 is preferentially expressed in lung cancer tissues compared with normal tissue and is implicated in the angiogenic stimulation in tumorigenesis [11,29]. Nowadays, the research interest has focused on the prognostic value of Ang-2 in lung cancer, while it has been proposed that Ang-2 can be regarded as an indicator of poor prognosis of NSCLC [16]. Moreover, a limited number of studies have evaluated the role of Ang-2 in SCLC cases [17,18]. In the present analysis, increased serum levels of Ang-2 were detected in lung cancer patients when compared with healthy controls, while higher baseline serum levels of Ang-2 were also identified in NSCLC and SCLC histological groups compared with healthy individuals, thus supporting the previous findings [16,17,18,25,26,27] (Table 2). No significant association of Ang-2 serum levels was identified when compared with the age, smoke, and gender of lung cancer patients (NSCLC, SCLC). It is of note that Ang-2 serum levels were not associated with metastasis in both SCLC and NSCLC incidences. With respect to NSCLC, Park et al. (2007) [16] first reported that high serum levels of Ang-2 are associated with metastasis in NSCLC patients. However, more recent analyses failed to confirm any significant association between serum Ang-2 and metastasis in NSCLC patients, which are in line with our results [14,25,30]. The impact of Ang-2 on metastasis in SCLC patients has not been investigated yet [17,18]. However, in the present analysis, we found no significant difference in Ang-2 serum levels between SCLC metastatic and non-metastatic cases, raising a concern about the prognostic significance of Ang-2 in the overall population.

Considering the prognostic role of Ang-2, numerous research studies have proved that high serum levels of Ang-2 are associated with poor prognosis in NSCLC [14,16,25,26,27]. On the other hand, a previous analysis by Naumnic et al. (2009) [30] demonstrated that the measurement of Ang-2, Ang-1, and Tie-2 serum concentration has no clinical impact on the prognosis of NSCLC. Our results indicate that higher baseline serum levels of Ang-2 are associated with poor prognosis in stage IIIβ NSCLC, while high baseline Ang-2 serum concentration is an independent factor of poor prognosis in stage IIIβ NSCLC (hazard ratio = 2.97, 95% CI: 1.05–8.40, *p* = 0.04, Table 3) [14,16,26,27]. Interestingly, no considerable association was detected between the serum levels of Ang-2 and the overall survival of stage IV NSCLC. In addition, we found that the serum levels of Ang-2 were not associated with the survival of SCLC patients, neither of stage IIIβ nor of stage IV. These results contradict previous findings that support that Ang-2 is a significant marker of poor prognosis in SCLC [17,18]. One possible explanation of our results could be the small number of SCLC patients examined in the present analysis. Thus, more analyses are warranted to better assess the prognostic capacity of Ang-2 in SCLC.

Finally, we investigated the alteration of Ang-2 serum levels after the third cycle of chemotherapy whenever patient serum samples were available. In particular, NSCLC patients were classified into two categories, containing those who received platinum-based chemotherapy and those who were treated with platinum-based chemotherapy in combination with an anti-VEGF factor. Ang-2 serum levels did not differ significantly after the third cycle of treatment compared with serum levels at admission in NSCLC and SCLC patients who received platinum-based chemotherapy (*p* = ns, Figure 5B) [17,18]. On the other hand, a considerable increase in Ang-2 concentration was detected in 16 NSCLC at the third cycle of treatment who received platinum-based chemotherapy in combination with the anti-VEGF treatment (*p* = 0.044, Figure 5A). Previous analyses have observed an increased Ang-2 expression as an escape mechanism to anti-VEGF therapy [31,32,33,34]. In clinical studies concerning glioblastoma, Ang-2 levels are reduced provisionally following inhibition of the VEGF pathway and eventually increased as tumors are turned into resistant to therapy [32]. Accordingly, vague resistance to anti-angiogenic therapy by VEGFA signaling blockade was found to be responsible for the upregulation of Ang-2 in late stage pancreatic neuroendocrine tumors [35]. Notably, it has been established that resistance to anti-VEGF treatment may include the adaptive enforcement of Ang-2/Tie2 signaling pathway and stimulate a reversal by Ang-2 neutralization in cases of pathological neovascularisation [36,37,38]. Taking all these data into consideration, it was concluded that the increase in Ang-2 in 16 NSCLC patients after three cycles of treatment with platinum-based chemotherapy and anti-VEGF might be related to the role of Ang-2 in resistance to anti-VEGF therapy.

It has been suggested that treatment with anti-VEGF factors improves the OS of NSCLC patients. The addition of avastin to atezolizumab and platinum-based chemotherapy offered a prolongation of OS from 14.7 months to 19.2 months (IMpower 150 trial), which led to the incorporation of anti-VEGF treatment in the first-line setting of NSCLC [39]. Independently of baseline Ang-2 levels, our results indicated that the incorporation of anti-VEGF factor into anticancer treatment prolonged OS, but it was not statistically significant affected. Our observations may be due to the small number of incidences that we examined in the present analysis. Therefore, more studies are required to draw more accurate conclusions, whereas the implication of Ang-2 as an escape mechanism to anti-VEGF therapy in NSCLC remains to be further discussed in the future.

The fact that the Ang-2 serum concentrations remain high even after the third cycle of chemotherapy either in NSCLC or SCLC cases may indicate that the respective angiogenic factor could serve as a considerable therapeutic target for lung cancer patients. Interestingly, the Ang-2 blockade decreases angiogenesis and inhibits metastasis in mouse tumor models, while the Ang-2 blockade restrains tumor growth and decelerates tumor resistance to anti-VEGFA therapy in various types of cancer when administrated in combination with anti-VEGF drugs [35,40,41,42,43,44]. Moreover, it has been proved that concurrent Ang-2 and VEGF blockade confers significant therapeutic benefits compared with the blockade of single agent in tumor models concerning metastatic breast cancer, pancreatic neuroendocrine tumor, and melanoma [35,45]. Taking all these data into consideration, it is proposed that Ang-2 might be an interesting target for future therapeutic approaches in lung cancer, while the therapeutic benefit of simultaneous blockade of Ang-2 and VEGF in lung cancer remains to be further clarified. In relation to SCLC, three cycles of chemotherapy resulted in an insignificant decrease in serum Ang-2 levels, which might be an indicator of stabilization of the disease. Additional samples at different time points could be useful in order to specify the time of relapse.

## 5. Conclusions

In conclusion, the present analysis revealed that Ang-2 is a significant marker of the development of NSCLC and SCLC. Moreover, our results indicate that the measurement of Ang-2 serum concentration has a considerable clinical role in the prognosis of stage IIIβ NSCLC, although this phenomenon was not observed in SCLC. The incorporation of Ang-2 in the diagnosis of stage IIIβ NSCLC could significantly improve the precise prognosis of lung cancer, while Ang-2 blockade could serve as a potential future therapeutic approach for lung cancer.

## Figures and Tables

**Table 1:** Association of Ang-2 baseline level with the different clinicopathological characteristics of patients including age, smoke, gender, metastasis, and histological type. *p*-values were considered as statistically significant at the 0.05 cut-off level.

	Ang-2 Median (pg/mL)	*p*-Values
Patients (n = 99)	3459	
Control (n = 55)	2148	<0.0001
Patients		
Age		
≥65 (n = 50)	3520	
<65 (n = 49)	3479	0.91
Smoke		
Yes (n = 62)	3643	
No (n = 37)	3338	0.16
Gender		
Males (n = 74)	3493	
Females (n = 25)	3526	0.61
Metastasis		
Metastasis (n = 64)	3538	
No Metastasis (n = 35)	3318	0.23
Lung cancer type		
NSCLC (n = 73)	3508	
SCLC (n = 26)	3479	0.91

**Table 2:** Association of Ang-2 baseline level in SCLC and NSCLC incidences with age, smoke, gender, and metastasis. *p*-values were regarded as statistically significant at the 0.05 cut-off level.

SCLC	Ang-2 Median (pg/mL)	*p*-Values	NSCLC	Ang-2 Median (pg/mL)	*p*-Values
Patients (n = 26)	3479		Patients (n = 73)	3508	
Control (n = 55)	2148	<0.0001	Control (n = 55)	2148	<0.0001
Patients			Patients		
Age			Age		
≥65 (n = 13)	3552		≥65 (n = 44)	3573	
<65 (n = 13)	2683	0.64	<65 (n = 29)	3350	0.96
Smoke			Smoke		
Yes (n = 20)	3434		Yes (n = 43)	3628	
No (n = 6)	3678	0.8	No (n = 30)	3161	0.24
Gender			Gender		
Males (n = 19)	3493		Males (n = 55)	3458	
Females (n = 7)	2683	0.4	Females (n = 18)	3538	0.9
Metastasis			Metastasis		
Metastasis (n = 19)	3891		Metastasis (n = 45)	3526	
No Metastasis (n = 7)	2683	0.11	No Metastasis (n = 28)	3350	0.61

**Table 3:** Multivariable analysis of the prognostic roles for clinicopathological parameters in patients diagnosed with stage IIIβ NSCLC.

Parameters	Hazard Ratio	Low Limit	Upper Limit	*p*-Value
Ang-2	2.97	1.05	8.40	0.04
Age	0.67	0.21	2.14	0.50
Smoke	0.96	0.34	2.71	0.94
Gender	0.76	0.22	2.69	0.68

## Data Availability

Data are included in the manuscript.
